# Video Education in Early Pregnancy and Parent Knowledge of Neonatal Resuscitation Options: A Secondary Analysis of a Randomized Clinical Trial

**DOI:** 10.1001/jamanetworkopen.2023.44645

**Published:** 2023-11-27

**Authors:** Siobhan M. McDonnell, Kathryn E. Flynn, Jennifer J. McIntosh, Ruta Brazauskas, U. Olivia Kim, S. Iqbal Ahamed, Mir A. Basir

**Affiliations:** 1 Department of Pediatrics, Medical College of Wisconsin, Milwaukee; 2 Department of Medicine, Medical College of Wisconsin, Milwaukee; 3 Department of Obstetrics and Gynecology, Medical College of Wisconsin, Milwaukee; 4 Division of Biostatistics, Medical College of Wisconsin, Milwaukee; 5 Department of Pediatrics, NorthShore University HealthSystem, Evanston, Illinois; 6 Department of Computer Science, Marquette University, Milwaukee, Wisconsin

## Abstract

This secondary analysis of a randomized clinical trial investigates the proportion of correct answers on neonatal resuscitation options among parents after seeing a video on these options.

## Introduction

For infants born during the periviable period of gestation, the decision to provide delivery room resuscitation is based on parental preference.^1^ Even when preterm birth risk factors (eg, chronic hypertension) are identified in early pregnancy, most parents are first offered information on neonatal resuscitation options upon periviable delivery hospitalization,^2^ when time constraints and stress limit contemplation. In the randomized clinical trial (RCT) of the smartphone Preemie Prep for Parents (P3) program,^3^ we provided neonatal resuscitation education in early pregnancy and evaluated the association of the program with knowledge at 25 weeks’ gestational age (GA).

## Methods

The Medical College of Wisconsin institutional review board approved this secondary analysis of an RCT, which followed CONSORT reporting guidelines (ClinicalTrials.gov Identifier: NCT04093492) (see trial protocol in Supplement 1). Pregnant patients with preterm birth risk factors were recruited from the high-risk obstetric clinic between February 2020 and April 2021 and randomized 1:1. The randomization sequence was generated using R statistical software version 3.6.0 (R Project for Statistical Computing) package blockrand and implemented through research electronic data capture (REDCap). Participants in P3 received text-messages with links to short, animated videos. Participants in the control group received patient education web pages from the American College of Obstetricians and Gynecologists. Participating partners were assigned to the same study group as the pregnant patient. Starting at 21 weeks’ GA, participants in P3 received 7 videos on neonatal resuscitation decisions (eg, Video; eTable in Supplement 2). At 25 weeks’ GA, assessments on neonatal resuscitation knowledge were collected via REDCap. Outcome assessors were blinded to study group. The trial ended when the predetermined sample size was reached.^3^

**Video.** Example Preemie Prep for Parents (P3) Video About Periviable Birth Treatment Options This example of a P3 video introduces the options for care in the delivery room after a periviable birth: using medical machines, using comfort care, or using limited medical machines.

In this post hoc, intention-to-treat analysis, the proportion of participants correctly answering each of 3 resuscitation questions was calculated in each study group. The difference in proportions is presented with the 95% CI.

## Results

Of 173 included participants (8 Asian [4.6%], 38 Black [21.9%], and 132 White [76.3%]; 18 Hispanic [10.4%]), most individuals were pregnant and had more than a high school diploma (Table; eFigure in Supplement 2). Among 120 pregnant patients, 1 individual delivered between 22 and 24 weeks’ GA. Of 94 participants in the P3 group, 87 individuals (92.6%) watched 1 or more of 7 resuscitation videos. There were 79 participants in the control group. Participants in the P3 group were more likely to know the parental role in resuscitation decisions, lower and upper GA limits when parents are asked to make resuscitation decisions, and all 3 neonatal resuscitation options available at our institution (Figure). For example, 55.6% of participants in the P3 group correctly identified all 3 resuscitation options compared with 4.1% of participants in the control group.

**Table.** Participant Demographics

Characteristic	Participants, No. (%)		
	Total (N = 173)	P3 group (n = 94)	Control group (n = 79)^a^
Parent type			
Pregnant patient	120 (69.4)	60 (63.8)	60 (75.9)
Partner	53 (30.6)	34 (36.2)	19 (24.1)
Race^b^^,^^c^			
Asian	8 (4.6)	3 (3.2)	5 (6.3)
Black	38 (21.9)	20 (21.3)	18 (22.8)
White	132 (76.3)	74 (78.7)	58 (73.4)
Other^d^	5 (2.9)	3 (3.2)	2 (2.5)
Ethnicity^b^			
Total with data, No.	172	93	79
Hispanic or Latino	18 (10.4)	10 (10.8)	8 (10.1)
Not Hispanic or Latino	154 (89.0)	83 (89.2)	71 (89.9)
Education			
≤High school diploma	27 (15.6)	12 (12.8)	15 (19.0)
Some college	57 (32.9)	36 (38.3)	21 (26.6)
4-y Degree	50 (28.9)	28 (29.8)	22 (27.8)
Graduate degree	39 (22.5)	18 (19.1)	21 (26.6)

^a^ Among 81 participants originally in the control group, 2 participants were excluded from analysis because they were support person participants who were not parents.

^b^ Race and ethnicity were self-reported by participants selecting among National Institutes of Health–specified racial and ethnic categories. Race options included American Indian or Alaska Native, Asian, Black or African American, Native Hawaiian or Other Pacific Islander, White, and other. Ethnicity options included Hispanic or Latino and not Hispanic or Latino. Race and ethnicity were reported because of past work demonstrating differences in neonatal intensive care at periviability by race and ethnicity.

^c^ Race percentages may not add to 100% given that participants could report being more than 1 race.

^d^ Other races included American Indian, Native Hawaiian or Other Pacific Islander, and self-selected other. Because each of these categories included 2 participants or fewer, these categories were summed and combined.

**Figure.**
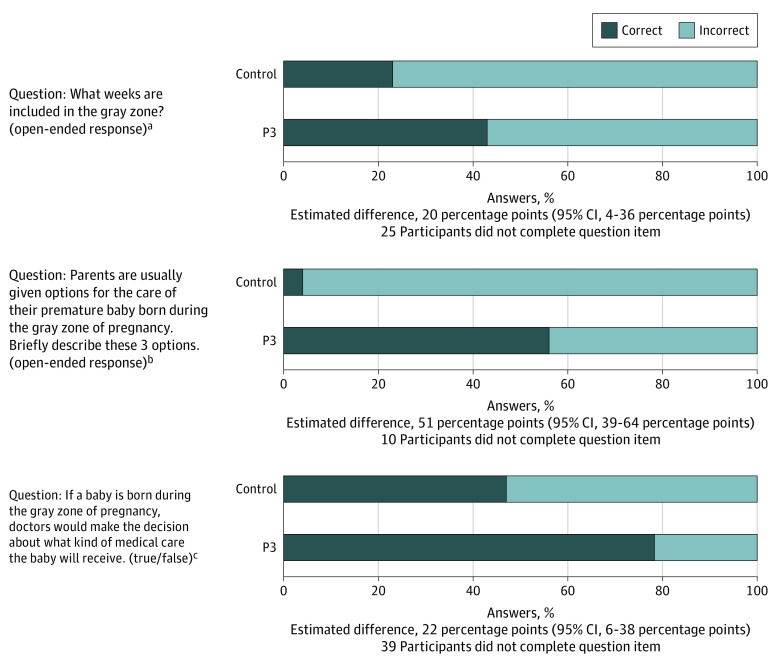
Participant Correct Answers on Knowledge Questions ^a^The correct response was 22, 23, and 24 weeks’ gestational age. ^b^The correct responses were medical machines, comfort care, and limited use of medical machines. ^c^The correct response was false.

## Discussion

P3 is a novel prenatal program that provides neonatal resuscitation education in early pregnancy to parents with preterm birth risk factors. In this secondary analysis of an RCT, most parents watched at least 1 video and video use increased parental knowledge.

Published research on improving the periviable decision-making process has focused on informing parents of neonatal resuscitation options at the time of delivery hospitalization.^4^ This situation is characterized by crucial information delivered with limited time for family discussion or contemplation. Although this time pressure may influence decisions,^5^ these limitations are assumed to be unavoidable. However, for pregnancies at risk of preterm birth, P3 videos provide parents with a framework of this decision and available options prior to hospitalization.

This study is limited in that findings were based on post hoc analysis. While our single-center trial was not intended to examine effects of knowledge on periviable resuscitation decisions (1 of 120 patients delivered at 22-24 weeks), already having foundational knowledge of options may allow counseling during hospitalization to focus more on personalized information and parental values.^6^

Whether to resuscitate an infant who is periviable and preterm is a complex, preference-sensitive decision. These results suggest that the P3 program may offer families with high-risk pregnancies the opportunity to learn about options available and contemplate what they would value for their baby before they may need to make the choice.

## Notes

Supplement 1.
Trial Protocol and Statistical Analysis Plan

Supplement 2. **eTable.** Neonatal Resuscitation Video Titles and Learning Objectives **eFigure.** Study Flowchart

Supplement 3.
Data Sharing Statement
