# Supplementary material for: Video Education in Early Pregnancy and Parent Knowledge of Neonatal Resuscitation Options: A Secondary Analysis of a Randomized Clinical Trial
Source: JAMA Netw Open. 2023 Nov 27;6(11):e2344645. doi: 10.1001/jamanetworkopen.2023.44645 (PMC10682831; doi:10.1001/jamanetworkopen.2023.44645)

STUDY RATIONALE

Background

Obstetric (ACOG) and Pediatrics (AAP) practice guidelines recommend that parents at risk for premature birth receive information regarding premature birth and potential health outcomes The NICHD 2014 Periviable Birth Joint Workshop concluded that the current process of providing information to parents at risk for premature birth needs improvements. Our proposal evaluates a new and substantively different approach to *when, where and how* prematurity education is provided to parents at risk for preterm birth. When: Anticipatory education is currently provided when the mother is in preterm labor, when time is short, and emotions run high. Our approach initiates education several weeks before the decision point. Where: Information is currently provided in the hospital, when families may be distracted or distressed and may not be together to discuss the information. Our approach provides information at home when parents can contemplate and discuss the information. How: Information is currently provided primarily verbally. Our approach provides multimedia information based on health literacy principles and developed by parents of preterm infants and experienced perinatal professionals.

Preliminary studies: Our previous work has informed the direction of the proposed work.

First, we prospectively assessed effectiveness of bedside verbal information by administering a questionnaire post-counseling to 49 women hospitalized for preterm birth. Notably, knowledge of short-term outcomes (lung immaturity, brain-bleed, infections, retinopathy, and nutrition) was higher (89% vs 53%) than knowledge of long-term outcomes (cerebral palsy, learning problems, behavior problems, chronic lung disease, blindness, and hearing impairment).

Second, we developed a printed handout to augment verbal information. In an RCT of 60 women hospitalized for preterm birth, the control group received verbal information from a clinician and the intervention group additionally received a printed handout. Knowledge of long-term outcomes was higher in the intervention group, (71% vs 45%, P=0.02). This evidence-based printed handout was recognized in the 2015 American Academy of Pediatrics (AAP) Periviability Practice Guidelines. Despite benefit in limited trials, printed information has failed to have a wider impact due to difficulties in dissemination and scalability.

We then developed the P3 mobile education as multimedia information improves learning and digital tools decrease logistic challenges. Obstetricians in our hospital system introduced the P3 education to 31 parents at the time of diagnosis of a risk factor for preterm birth. The P3 mobile education was downloaded to the participant smartphone from Apple store or Google play. The median (range) GA at enrollment was 20 (18-22) weeks. Four weeks after enrollment, 28 participants (18 mothers and 10 fathers) completed the final survey. GA at birth is known for 15 of the 18 mothers, with 6 (40%) experiencing preterm birth between 22-36 weeks GA. Among the 15 participants for whom utilization data is available, over the 4 weeks of participation, the P3 mobile education was accessed for a total of 118.5 hours, with range of 6 minutes to 14 hours per participant. Participants reported receiving more information from the P3 mobile education than from their healthcare provider, in the areas of preterm labor, premature infants and parental role and responsibilities.

Select survey items and participant responses	Yes (%)
**Medical knowledge**	
1. Did the app provide new information to you?	100
2. Was some of the information given in the app important for you to know?	100
3. Because of the app, do you feel better prepared if you were to have a premature infant?	100
4. In your opinion, will this app benefit families?	100
5. Did the app inform you about difficulties that a premature baby may face at birth?	89
**Emotional health**	
1. Did the app make you feel more aware of your risk for having a premature delivery?	93
2. Did the app increase your anxiety unnecessarily?	14
3. As time passed and you were familiar with the information provided, did the app increase your anxiety about having a premature infant later?	21
**Partnership and Advocacy**	
1. Because of the app, did you ask more questions at your doctor visits?	43
2. Because of the app, did you and your partner discuss pregnancy or prematurity issues?	86
3. Did you share information given in the app with other family/friends (excluding a partner already enrolled in the study)?	57

Theoretical frameworks and hypotheses. Cognitive load theory suggests that an individual's capacity to process information is finite and limited by the individual's initial processor of information referred to as "working memory." The Dual Code Theory suggests that humans have separate working memory for auditory/text information and for visual information. Using both routes increases uptake of information. The Theory of Multimedia Learning states that even when cognitive load is not an issue, people will learn more with a blend of combined media than from a single medium. Finally, according to the Health Belief Model, health behavior (decisions) stem from beliefs (knowledge), perceived benefits of and barriers to action, and self-efficacy.

Thus, **we hypothesize** that the multimedia P3 mobile education will result in 1) higher parental knowledge and 2) better preparation for decision making compared to a static, electronic ACOG educational handout.

STUDY APPROACH

Sample

Participants will be recruited from the obstetric clinics at the Froedtert Lutheran Memorial Hospital (FLMH), which is the adult tertiary care hospital co-located on the Milwaukee County campus with Children's Hospital of Wisconsin. FLMH is the primary teaching hospital for the Medical College of Wisconsin and it has a multi-state referral base, a high-risk pregnancy center, fetal intervention program, and a fertility clinic.

<u>Inclusion criteria</u>: Eligible participants will be (**1**) pregnant women, including pregnant minors aged 13 and above, (**2**) with presence of one of the following maternal preterm birth risk factors: history of spontaneous preterm birth, shortened cervical length, multiple gestation, fetal growth restriction, chronic hypertension, history of preeclampsia, and/or diabetes requiring medications, (**3**) with fetal GA between 14-21 weeks at enrollment to allow parents time to review the study information before birth, while also approaching a GA when neonatal resuscitation is an option (22 weeks), (**4**) who have a smartphone, and (**5**) who are able to read and speak English (the P3 mobile education is currently only available in English). For all women (or pregnant teenagers) who are eligible and agree to participate, we will invite their partner to participate; however, partner participation is not required. Partner is defined as the mother's primary pregnancy support person; in the majority of cases this is the father of the baby.

<u>Exclusion criteria:</u> Pregnancies with known significant birth defects will be excluded as information provided to these parents is different.

Aim 1: Determine the effect of the P3 app on NICHD-recommended parental prematurity knowledge.

Study procedure (Please see Appendix A):

<u>Pre-enrollment preparation</u>.

1) The study team will be trained by a perinatal psychologist (Dr. Abbey Kruper, see Letter of Support) to recognize participant anxiety and address or refer appropriately.

2) Tutorials will be provided to familiarize prenatal obstetric providers with the study educational-aids, inclusion/exclusion criteria, and other study procedures.

3) P3 mobile education functionality will be upgraded based on feedback from the previous pilot testing. Dr. Ahamed's UBITRIX team will incorporate the suggested changes. A review of the upgraded content based on the principles of health literacy will be performed by Dr. Barnekow (Families First LLC).

4) Usability testing of the upgraded materials will be conducted with the general population, to determine users' understanding of specific words and concepts in the videos. Please see PRO 34774 for more information. Some findings of the usability testing may not be able to be changed within the scope of this project, but recommendations will be provided on work-arounds or changes that could be made in the future.

<u>Enrollment and consent</u>.

1) The clinical research assistant (CRA) will screen daily obstetric clinic appointments to identify potentially eligible parents.

2) The obstetric provider will introduce the study to eligible parents and refer interested parents to the CRA.

3) The CRA will meet with the parents and invite all eligible pregnant women (or minors) and present partners to participate. For partners who are not present at the time of the obstetric clinic appointments, the pregnant women (or minors) will be asked if they have a support person for this pregnancy. If so, they are asked if they would be willing to provide this person's contact information. If they offer the information, these partners will be contacted and invited to join the study by the CRA, with consenting process through REDCap.

4) As part of the consent process, parents will be provided detailed study information, the risks to confidentiality, the risk of heightened anxiety, the extent of researcher access to health records, as well as what will happen to data already collected should the participant withdraw from the study. Parents who decline participation will continue to receive standard pregnancy and prenatal care. Participants will also be asked during the enrollment process if the CRA can add P3 study as a contact in their phone, so that the CRA office number, the study phone number, and the study email are in the participants' phone.

Randomization

1) Pregnant women (or minors) who consent to participate will be randomized 1:1 to the P3 mobile education (intervention) or the link to electronic ACOG handout arm (active control), with assignment to be determined by REDCap displaying the computer-generated random assignment. Block randomization will be used with random block sizes of 2 and 4, based on the gestational age of the pregnant woman at the time of enrollment, with participants with baseline assessment during weeks 16-18 in one block, and those with baseline during weeks 19-21 in another block. A randomization list will be generated using the R package blockrand

2) Eligible partners who consent to participate will be assigned to the same intervention group as the pregnant participant, otherwise they will contaminate each other.

3) Participants will receive either links to ACOG education or be added as a user to the P3 mobile education site via email or text to their smartphone. The CRA will then provide a brief tutorial and the study team contact information.

Study Groups

P3 mobile education group

Participants will then receive a schedule of automated text messages, with video links. These videos and messages are based on information relevant to their gestational age. The messages will be sent a maximum of two times per day. If participants do not click on the link after 4 hours, they may receive a reminder message.

The participants from this group will also be able to directly access the P3 website, where the videos and educational materials are housed, to use the educational videos if they wanted to “binge watch”.

The P3 mobile education group participants use of educational materials will be tracked on the HIPPA complaint P3 website. The site will not house any PHI information and each participant use of the site will be tracked using their study assigned number. Tracked information will include clicking on the video link, which of the educational videos were watched, and for how long.

Based on our tracking data, if there evidence of a technical malfunction in our text-video link function, such as the participant has only seen 10% or less of the videos texted to them, the CRA may follow up with participants via phone or email to make sure they are still able to receive messages and access the videos.

ACOG electronic handout group

Participants will receive links to ACOG patient premature birth education related handouts

Data collection. (Please see Survey Schedule in Section 52.)

At baseline (16-21 weeks) the CRA will administer the *health literacy assessment*, will ask participants to self-administer the *Entry Questionnaire*, an *anxiety measure*, and a subjective *global health measure*, and will abstract *maternal clinical data* and *reproductive history* from the medical record.

As pregnancy continues, REDCap will automatically send out questionnaires to participants at key time points: the start of their 25th, 30th, and 34th weeks. These times will include *Prematurity Knowledge Questionnaires* (specific to their gestational age), a *Preparation for Decision Making scale*, and an *anxiety measure*. At these time points, if they do not complete the questionnaire the first day they are sent it, they will

be sent an automatic reminder through REDCap for the next 2 days. If the participant still has not completed the questionnaire by this time, the CRA will contact the participant by phone.

At study completion, within two weeks of either preterm childbirth or completion of the 34 week questionnaire, participants will be asked to also complete the *Educational-Aid Questionnaire*.

Participants will receive $20 for each completed follow-up assessment (up to $80) and entered into a lottery to win an additional $50 for every questionnaire they return the day they were sent it.

Data will be collected and managed using the REDCap (Research Electronic Data Capture) system. This system is a secure, web-based application designed to support data collection for research. It provides data validation, audit trails, and automated export procedures to statistical packages. Branching logic and calculated fields can also be created in the system to support data entry.

Study Measures

Apart from the Newest Vital Sign, all measures will be self-administered by participants.

The *Newest Vital Sign* is a validated health literacy scale that takes 3 minutes for a trained interviewer to administer. The participant is given a special ice cream label to review and is asked questions (by the CRA) to test understanding of words and numbers. Because it cannot be self-administered, this tool will only be used for the pregnant participant, as we know they will be in-person for intake.

The *Brief Health Literacy Screening* is a validated health literacy screening that in only one question is able to assess health literacy levels. This tool will be used with the partner participant, as it can be self-administered (unlike the Newest Vital Sign) and partners may not be present at the obstetrician appointment to enroll in-person.

The PROMIS Global Health scale is a measure of physical, mental, and social health. Its ten items are subjective self-report, and as such will be given to both the pregnant woman and her partner, though we have some information about the pregnant participants' health from her medical record.

The *Entry-Questionnaire* will collect information regarding socio-demographics, mobile technology use, family relationships, and participant's decision-making values.

The *Prematurity Knowledge Questionnaire* assesses knowledge of the information recommended by the NICHD panel for periviable birth.[1] It consists of following knowledge domains: due date estimation, lowest GA necessary for survival, factors influencing preterm birth outcome, treatment options at periviable GA, long-term outcomes, short-term outcomes, and advocacy. It consists of a combination of fill-in-the-blank, multiple choice, and true/false questions. The items were evaluated and revised through cognitive interviews, which rely on intensive verbal probing of participants by a trained interviewer to identify otherwise unobservable problems with item comprehension, recall, and other cognitive processes that can be remediated through question rewording, reordering, or more extensive revision..

The *Educational-Aid Questionnaire* will gather user feedback regarding the assigned educational-aid, including whether the educational aid increased awareness of risk of preterm birth, influenced communication with the obstetric providers, influenced pregnancy communication with partner, whether their doctor or the educational-aid provided more prematurity information, ease of use, and encouraged parents to learn more information. The questionnaire for the P3 group has additional items regarding audiovisuals and the look of the app.

Sample Size

Based on our published data for knowledge of long-term outcomes, we expect average parent knowledge of seven long-term outcomes of 50% (SD 35%) in the ACOG group. In our population, English literacy is ~90% while ~80% possess smartphones. With 50 participants in each group, we can detect a 20% difference in knowledge, with power of 80% and α of 0.05. To allow for 10% attrition, 55 pregnant participants will be enrolled in each group.

Recruitment Feasibility

Our institutional Clinical Research Data Warehouse 2016 clinic patient population shows: 303 pregnancies with hypertension and preeclampsia, 302 with fetal growth restriction, 218 with multiple gestations, 87 with shortened cervix, 80 with history of preterm labor, 68 with pre-existing diabetes, and 23 with cerclage. These

numbers indicate that goal recruitment is unlikely to be a problem. For the proposed study, we plan to enroll ~10 women (or minors) per month to reach our target enrollment within 1 year.

Data Management.

All data will be stored securely at the Medical College of Wisconsin in REDCap. Personal identifiers will be kept separately, and only key personnel will have access to the identifiers. The P3 site is HIPPA compliant; however, we will not have PHI information on the P3 site as participants study ID numbers will be used.

Data Analysis

Dr. Brazauskas will perform 1) blinded scoring of the Prematurity Knowledge Questionnaire using prespecified rules for each response, and 2) blinded analysis with masked group assignment in the dataset. The randomized design, the blinded scoring, and the blinded comparison of post-intervention NICHD recommended knowledge between the P3 and ACOG groups should achieve robust and unbiased results. The Educational-Aid Questionnaire responses and the P3 tracking data will be analyzed separately after completion of the other analyses.

Statistical analyses will be conducted in SAS. The primary outcome is the knowledge score for long-term outcomes (cerebral palsy, learning problems, behavior problems, chronic lung disease, blindness, and hearing impairment), and the primary analysis will use Student's t-test to compare the two groups on knowledge of long-term outcomes at 25 weeks. We will use regression methods to examine relationships between knowledge and sociodemographic characteristics, health literacy level, and the mobile intervention use pattern captured by the P3 software. Additional planned analyses will compare knowledge at later time points and in the other NICHD-recommended domains, e.g. short-term outcomes, as well as differences between groups in preparation for decision-making scores and anxiety scores. We will also use regression methods to examine relationships between knowledge and sociodemographic characteristics, health literacy level, and the mobile intervention use pattern captured by the P3 software.

Aim 2: Determine the effect of the P3 mobile app on parental preparation for decision-making.

Study procedure, participants, and data management are the same as described for Aim 1.

Data collection. Participant decision self-efficacy will be assessed at study entry and exit. Preparation for decision-making and anxiety will be in conjunction with the knowledge questionnaire at 25, 30 and 34 weeks.

Study measures

Decision Self-Efficacy Scale is an 11-item scale that measures self-confidence or belief in one's abilities in decision-making, including shared decision-making.[19]

Preparation for Decision Making scale is a 10-item scale that assesses how useful a decision-support intervention is in preparing the respondent to communicate with their clinician about a health decision. It has been shown to discriminate between patients who did and did not find a decisional aid helpful with a difference of 0.6 points on a 1-5 scale, and SD ≈1.0.[21] It can be customized to represent the particular intervention and decision under consideration; in this case, decisions about resuscitation, hospital choice, and breastfeeding are focused on during appropriate time points.

PROMIS Anxiety Computerized Adaptive Test (CAT) measures general anxiety over the past seven days.[20] With CAT administration, participant responses guide the system's choice of subsequent items to enable a highly precise score with just ~5 items answered for most participants. PROMIS measures have evidence for validity in the general population and provide a T-score, where 50 (SD 10) corresponds to the US average.

Sample size

The analytic sample size of 100 provides 80% power to detect a difference of 0.6 points in Preparation for Decision Making (using SD=1.0), which was previously shown to be a meaningful difference for this scale. With 50 partners enrolled, a within-couple difference in Preparation for Decision Making of 0.4 points will be detectable with 80% power if the within-couple correlation is 0.5 or higher.

Data analysis

Preparation for decision making mean scores will be compared post-intervention at each time point across the two groups using Student's T-test. Associations between participant preparation scores, their level of domain specific knowledge, and socio-demographics will be explored using regression analyses. The primary analysis

will be focused on the pregnant women (or minors), with secondary analyses comparing between partners, and partners and women (or minors) within couples using paired T-tests.

Addressing Missing Data.

Despite our best efforts to prevent study dropout and missing data, we anticipate there will be some study dropout. If one member of a couple decides to drop out, we will try to retain the other member. We will attempt to describe differences among those who do and do not drop out, with the goal of characterizing the uncertainty created by missing data. We will explore whether certain characteristics are more prevalent among participants who drop out, e.g., high anxiety or neonatal death.

POTENTIAL CHALLENGES AND ALTERNATIVE STRATEGIES

Our Clinical Research Data Warehouse information suggests 12 months to enroll 110 participants will be sufficient. We will assess recruitment rates after 6 months, and if insufficient, then our neighboring partner perinatal centers can be added. We will attempt to collect all data in person in conjunction with participants' scheduled prenatal visits. In order to minimize missing data, if we have been unable to connect with a participant during a prenatal visit, we will allow self-administration through an emailed link to REDCap. Thus, there is a potential that a so-motivated parent may "cheat" in order to answer questions correctly. For all participants, we will stress at study enrollment and before each assessment that they should not expect to know all of the answers and that the point of the study is for us to understand how to improve the educational tool, thus their honest answers will be most helpful. We will conduct sensitivity analyses to evaluate knowledge scores based on in-person vs. at home administration.

EXPECTED RESULTS AND RESULTS DISSEMINATION

We plan a multi-faceted dissemination approach including presenting at meetings, articles in professional journals, and targeted dissemination to patient and physician groups. Manuscripts describing study results will correspond to Aims 1 and 2.

Appendix A: Flow of Study

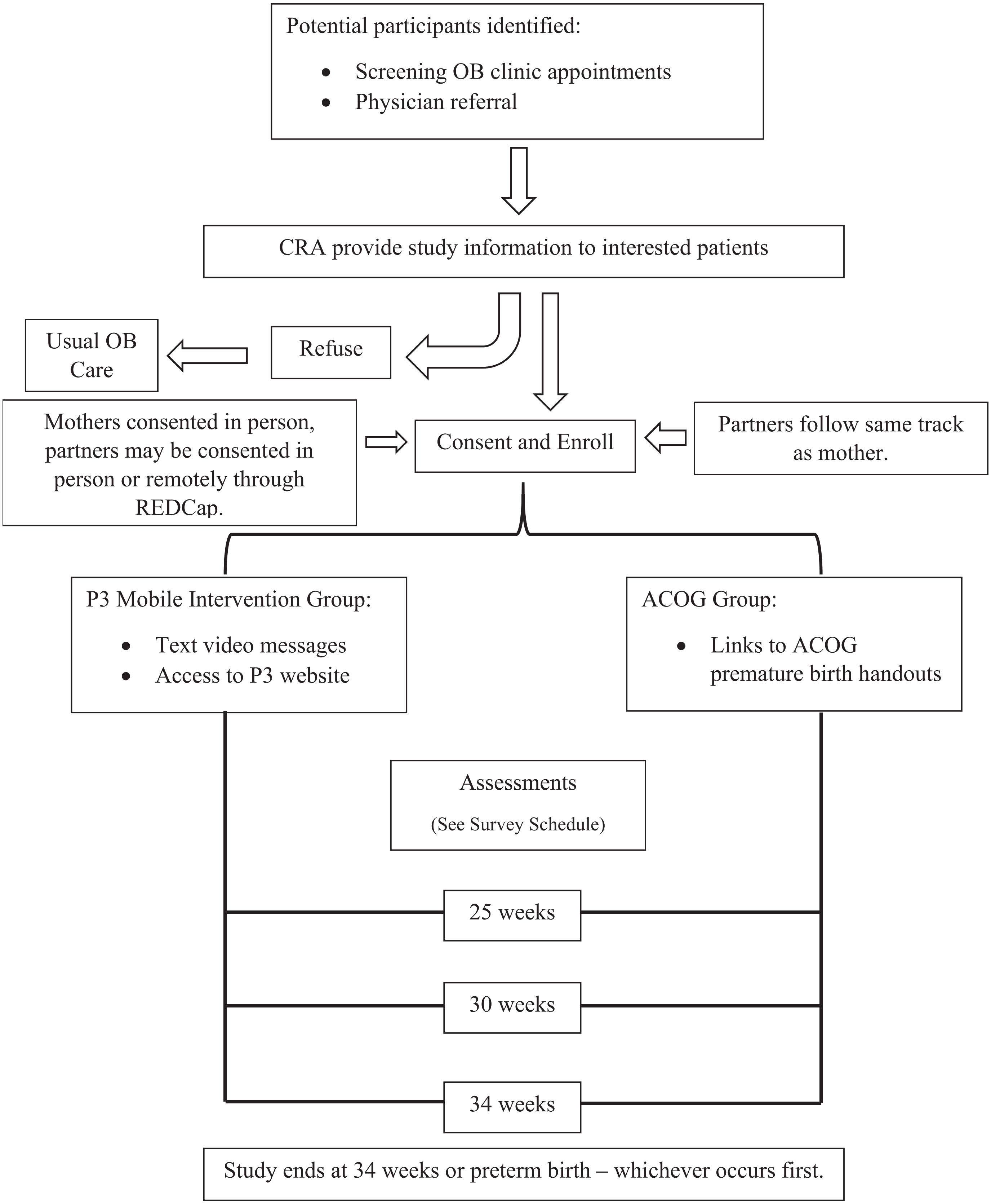

Supplement: Supplement 1. — Trial Protocol and Statistical Analysis Plan [file jamanetwopen-e2344645-s001.pdf]
