# Supplementary material for: Video Education in Early Pregnancy and Parent Knowledge of Neonatal Resuscitation Options: A Secondary Analysis of a Randomized Clinical Trial
Source: JAMA Netw Open. 2023 Nov 27;6(11):e2344645. doi: 10.1001/jamanetworkopen.2023.44645 (PMC10682831; doi:10.1001/jamanetworkopen.2023.44645)
Supplement: Supplement 2. — eTable. Neonatal Resuscitation Video Titles and Learning Objectives eFigure. Study Flowchart [file jamanetwopen-e2344645-s002.pdf]

Supplemental Online Content

McDonnell SM, Flynn KE, McIntosh JJ, et al. Video education in early pregnancy and parent knowledge of neonatal resuscitation options. *JAMA Netw Open*. 2023;6(11):e2344645. doi:10.1001/jamanetworkopen.2023.44645

eTable. Neonatal Resuscitation Video Titles and Learning Objectives

eFigure. Study Flowchart

This supplemental material has been provided by the authors to give readers additional information about their work.

© 2023 McDonnell SM et al. *JAMA Network Open.*

eTable 1. P3 program's neonatal resuscitation video titles and learning objectives.

Video Title	Learning Objective
1. Boundaries of Survival	Infants born before 22 weeks GA[a] are currently not able to survive.
2. The Gray Zone	At 22, 23, and 24 weeks GA[a] ("the gray zone"), parents typically decide between resuscitation options.
3. The 3 Treatment Options	During the gray zone, resuscitation decisions can involve medical machines, comfort care, or limited use of machines.
4. Option: Medical Machines	Resuscitation with medical machines involves use of breathing machines and CPR[b] but does not guarantee the baby will survive.
5. Option: Comfort Care	Comfort care is using warmth, physical touch, and medications to ensure the baby dies peacefully.
6. Option: Limited Machines	Resuscitation with medical machines can have limits depending on how the infant is responding, agreed on ahead of time.
7. If You Change Your Mind	If parents choose medical machines and then change their mind, they can typically ask physicians to stop.

[a] Gestational age
[b] Cardiopulmonary resuscitation

© 2023 McDonnell SM et al. *JAMA Network Open.*

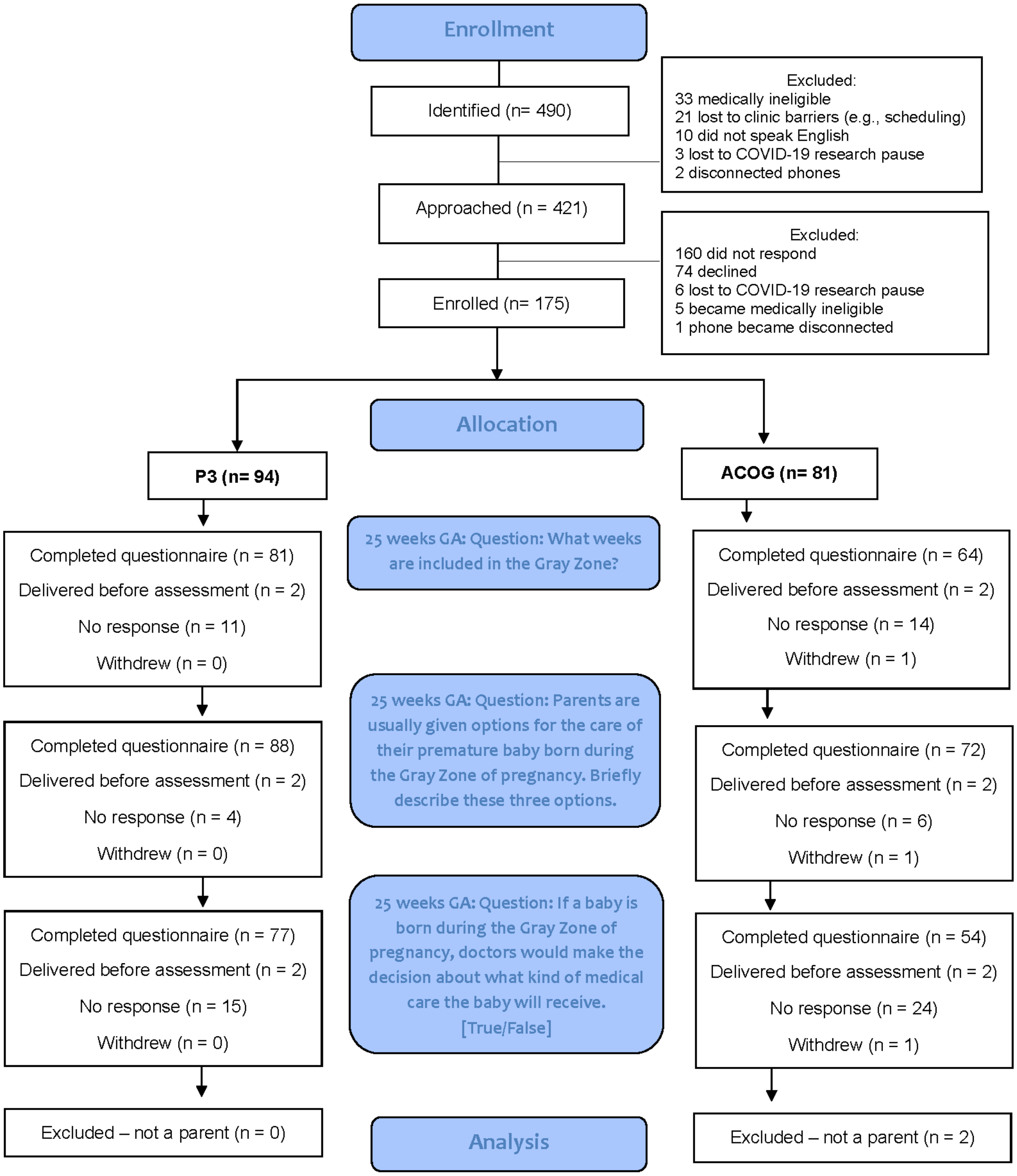

© 2023 McDonnell SM et al. *JAMA Network Open.*
