# Supplementary material for: Video Education in Early Pregnancy and Parent Knowledge of Neonatal Resuscitation Options: A Secondary Analysis of a Randomized Clinical Trial
Source: JAMA Netw Open. 2023 Nov 27;6(11):e2344645. doi: 10.1001/jamanetworkopen.2023.44645 (PMC10682831; doi:10.1001/jamanetworkopen.2023.44645)
Supplement: Supplement 3. — Data Sharing Statement [file jamanetwopen-e2344645-s003.pdf]

Data Sharing Statement

McDonnell. Video Education in Early Pregnancy and Parent Knowledge of Neonatal Resuscitation Options: A Secondary Analysis of a Randomized Clinical Trial . *JAMA Netw Open*. Published online November 27, 2023. doi:10.1001/jamanetworkopen.2023.44645

Data
Data available: No
